# Nanofibrous patches for targeted therapy of cutaneous leishmaniasis caused by *Leishmania major*: a preclinical amphotericin B platform

**DOI:** 10.1007/s00436-025-08605-x

**Published:** 2025-12-11

**Authors:** Mahya Allahmoradi, Mehdi Mohebali, Hamed Mirjalali, Mahdi Adabi, Fahimeh Firouzjaei Karder, Seyed Mahdi Rezayat, Abbas Rahimi Foroushani, Seyyedeh Elaheh Mousavi, Elham Kazemirad

**Affiliations:** 1 Department of Medical Parasitology and Mycology, School of Public Health, Tehran University of Medical Sciences, Tehran, Iran; 2 Center for Research of Endemic Parasites of Iran (CREPI), Tehran University of Medical Sciences, Tehran, Iran; 3 Foodborne and Waterborne Diseases Research Center, Research Institute for Gastroenterology and Liver Diseases, Shahid Beheshti University of Medical Sciences, Tehran, Iran; 4 Department of Medical Nanotechnology, School of Advanced Technologies in Medicine, Tehran University of Medical Sciences, Tehran, Iran; 5 Department of Pharmacology, School of Medicine, Tehran University of Medical Sciences, Tehran, Iran; 6 Department of Epidemiology and Biostatistics, School of Public Health, Tehran University of Medical Sciences, Tehran, Iran

**Keywords:** Amphotericin b, Cutaneous leishmaniasis, Electrospinning, Leishmania major, Nanofiber patch, Topical drug delivery

## Abstract

Cutaneous leishmaniasis (CL) remains a significant public health challenge in endemic regions, particularly where access to safe and patient-friendly treatments is limited. Amphotericin B (AmB), although highly active against *Leishmania*, is restricted by systemic toxicity and the need for parenteral administration. In this study, AmB-loaded bioactive nanofibrous patches were fabricated using a dual-nozzle electrospinning method incorporating chitosan, gelatin, and polyvinyl alcohol. The resulting nanofibers were structurally characterized by scanning electron microscopy, Fourier-transform infrared spectroscopy, and X-ray diffraction. Drug loading was uniform, and in vitro release demonstrated a sustained profile with approximately 82% cumulative release at 72 h. Cytocompatibility was confirmed in human dermal fibroblasts and THP-1 cells. The therapeutic performance was further assessed in BALB/c mice using short- and long-term treatment protocols. AmB-loaded patches produced a significant reduction in lesion size compared with untreated and placebo groups, and the treatment outcome was comparable to Glucantime^®^ (Day 21) and SinaAmpholeish^®^ (Day 63). No local irritation or observable systemic toxicity was noted. Overall, these findings indicate that nanofiber-mediated topical delivery of AmB provides a promising localized treatment approach for CL and warrants further investigation in translational and clinical studies.

**Supplementary Information:**

The online version contains supplementary material available at 10.1007/s00436-025-08605-x.

## Highlights

Dual-nozzle nanofibrous patches enable localized delivery of amphotericin B.

Uniform bioactive fibers support controlled and sustained drug release.

Amphotericin B release shows a stable sustained-release profile.

Nanofiber-based delivery reduces acute cytotoxicity compared with free drug.

Topical AmB patches improve lesion outcomes in a BALB/c CL model.

**Supplementary Information:**

The online version contains supplementary material available at 10.1007/s00436-025-08605-x.

## Introduction

Cutaneous leishmaniasis (CL) is a neglected vector-borne disease with considerable global impact, particularly in the Middle East, North Africa, and parts of South America and Central Asia. Among the causative species, *Leishmania major* and *L. tropica* are the predominant etiological agents in the Old World, including Iran, which remains a major endemic country (Firooz et al. 2021; Razavi et al. 2021; Sasidharan and Saudagar 2021).

According to the World Health Organization (WHO), approximately 600,000 to 1 million new cases of CL occur annually, with more than 87% reported in just ten countries (Sharifi et al. 2023; World Health Organization 2023). Meglumine antimoniate (Glucantime^®^) has been recognized as the first-line treatment for cutaneous leishmaniasis since the 1940 s and continues to be widely used in many endemic regions. However, its clinical application is increasingly limited due to severe side effects—including nephrotoxicity, local irritation, systemic inflammation—and the need for prolonged parenteral administration (Oliveira-Ribeiro et al. 2021). Moreover, rising drug resistance has led to changes in its recommended use in some countries (Fernández et al. 2024). For instance, a case report from Pakistan documented a 24-year-old male with multiple non-healing ulcers, indicative of cutaneous leishmaniasis, who did not respond to Glucantime^®^ treatment (Sultan et al. 2025).

Additionally, molecular studies have identified mutations in the Cystathionine β-synthase (CβS) and Ornithine decarboxylase (ODC) genes, which are used as resistance markers in resistant strains (Sundar et al. 2019; Zarrinkar et al. 2025). CβS is involved in the methionine and cysteine metabolism pathway, while ODC plays a key role in polyamine biosynthesis; mutations in these genes are associated with reduced drug susceptibility. These challenges often lead to incomplete treatment, increased healthcare burden, and the development of drug resistance (Croft et al. 2006; Moncada-Diaz et al. 2024). As such, alternative treatment modalities with better safety profiles and improved patient compliance are urgently needed. Amphotericin B (AmB), a broad-spectrum antifungal and antileishmanial agent, has been utilized as a second-line treatment for mucocutaneous and cutaneous leishmaniasis, particularly in antimonial-resistant cases (Roatt et al. 2020; Shirzadi 2019). The conventional formulation of AmB is administered parenterally (intravenous or intramuscular), which is limited by poor solubility, rapid degradation, systemic toxicity, and low bioavailability (Gonzalez et al. 2008; Layegh et al. 2011). In recent years, topical formulations and nanocarrier-based delivery systems—including liposomal and polymeric nanofiber patches—have been developed to enhance local efficacy, reduce systemic exposure, and improve patient compliance. These topical formulations allow for targeted delivery to cutaneous lesions, providing an ideal context for comparison with parenteral AmB in terms of both safety and therapeutic outcomes.

In recent years, nanotechnology has offered innovative approaches to overcome these limitations by enabling controlled drug delivery, reducing toxicity, and enhancing target specificity (De Santana et al. 2022; Mehrizi et al. 2018). Electrospun nanofibers, in particular, have demonstrated exceptional promise in transdermal and wound-associated drug delivery due to their high surface area, tunable porosity, and structural resemblance to the extracellular matrix (Asgari et al. 2021; Monge-Maillo and López-Vélez 2013).

While such systems have been widely explored in conventional wound models, it is important to note that cutaneous leishmaniasis (CL) lesions differ fundamentally from generic wounds. Unlike clinically “clean” or variably contaminated wounds, CL lesions are consistently associated with inflammatory cell infiltration, *Leishmania* amastigotes, and in some cases concomitant microbial infections. Therefore, the nanofiber platforms described here are considered particularly relevant, as their combined antimicrobial, immunomodulatory, and regenerative properties may offer advantages within the complex pathological microenvironment of CL.

These features not only improve drug loading and release kinetics but also support tissue regeneration and wound healing. Polymeric nanofibers made from chitosan, gelatin, and polyvinyl alcohol (PVA) have emerged as favorable platforms for dermal applications. Chitosan is known for its antimicrobial and biocompatible properties; gelatin promotes cell adhesion and angiogenesis; and PVA offers excellent mechanical strength and electrospinnability (i.e., the ability of a polymer solution to be stretched and continuously drawn into nanofibers under an electrostatic field) (Hodge et al. 2022; Xue et al. 2019).

A study has reported the successful incorporation of AmB into various nanocarriers, including chitosan nanoparticles and electrospun core-shell nanofibers (Asgari et al. 2021), showing improved efficacy against *Leishmania* parasites and reduced cytotoxicity. However, there remains a lack of comprehensive preclinical evaluation of electrospun AmB-loaded nanofibers, particularly in *L. major* models.

To address this gap, the present study aimed to fabricate and characterize electrospun chitosan/gelatin/PVA nanofibers loaded with AmB, and to evaluate their therapeutic efficacy in vitro and in vivo against *L. major*. In this study, the nanofibers loaded by AmB were assessed for morphological, mechanical, and physicochemical properties, drug loading and release profiles, cytocompatibility on both HDF and THP-1 cell lines. The antileishmanial activity in a BALB/c murine model was also determined. This work contributes to the development of safer, more effective, and patient-friendly alternatives for the treatment of CL.

## Materials and methods

### Study design and setting

This experimental study was conducted at the School of Advanced Technologies in Medicine, Tehran University of Medical Sciences (TUMS), between 2023 and 2024. The objective was to evaluate the in vitro and in vivo efficacy of amphotericin B (AmB)-loaded chitosan/gelatin/polyvinyl alcohol (PVA) nanofibers against *Leishmania major* using both cell culture and murine infection models. A total of 84 male BALB/c mice (*n* = 12 per group) were randomly assigned to seven experimental groups: Group 1 (Positive Control): Glucantime^®^ (100 mg/kg), administered intraperitoneally once daily for 14 days. Group 2: SinaAmpholeish^®^ 0.4% topical liposomal AmB ointment (Eskandari et al. 2019), applied twice daily for 56 days. Group 3: AmB-loaded chitosan/gelatin/PVA nanofiber patch, applied every other day for 14 days. Group 4: AmB-loaded nanofiber patch, applied once weekly for 56 days. Group 5: Blank (drug-free) nanofiber patch, applied every other day for 14 days. Group 6: Blank nanofiber patch, applied once weekly for 56 days. Group 7 (Negative Control): Untreated group monitored throughout the 63-day study period.

### Ethical approval

All animal procedures followed the guidelines of the Institutional Animal Care and Use Committee (IACUC). The study protocol was approved by the Ethics Committee of the School of Public Health, TUMS (Protocol No. IR.TUMS.AEC.1401.043). A total of 16 animals were euthanized based on predefined humane endpoints, including severe ulceration, persistent lethargy, marked weight loss, or lesion sizes exceeding 20 mm². Euthanized animals included 6 from Group 7 (Negative Control), 4 from Group 1 (Positive Control), 3 from Group 3 (AmB-loaded nanofiber; every other day), and 3 from Group 5 (Blank nanofiber; every other day). Detailed information is provided in Supplementary Table S1.

## Materials

Low molecular weight chitosan (Mw = 50,000–190,000 Da) was obtained from Chitotech (Tehran, Iran). Polyvinyl alcohol (PVA, Mw = 72,000 Da), edible bovine gelatin, and glacial acetic acid were purchased from Merck (Germany). Amphotericin B (AmB) was sourced from Xgen Pharmaceuticals (Big Flatts, NY, USA), while Glucantime^®^ was acquired from Sanofi-Aventis (France). Penicillin and streptomycin were purchased from Tehran Pharmacy (Iran). Fetal bovine serum (FBS; heat-inactivated, Gibco, Thermo Fisher Scientific, USA), RPMI-1640 medium supplemented with L-glutamine, and Tween 20 were obtained from Merck (Darmstadt, Germany). MTT powder (3-[4,5-dimethylthiazol-2-yl]−2,5-diphenyl tetrazolium bromide) was purchased from Biobasic (Ontario, Canada). All reagents and solvents used in the study were of analytical grade and used as received.

### Preparation of electrospinning solutions

Two distinct polymer solutions were prepared for random blending-type dual-nozzle electrospinning. The first solution was an aqueous acidic blend of chitosan (2% w/v) and gelatin (10% w/v) dissolved in 50% (v/v) acetic acid, based on extensive preliminary optimization to ensure electrospinnability, solution stability, and fiber uniformity. Among the initially tested weight ratios (0:100, 20:80, and 80:20), the 20:80 chitosan-to-gelatin ratio yielded the most consistent, bead-free fibers and was thus selected for all subsequent experiments. The pH of the solution was adjusted to 4.5 using 1 M NaOH, preserving the solubility of chitosan and structural integrity of gelatin. The second solution consisted of 10% (w/v) polyvinyl alcohol (PVA; Mw = 72,000 Da) dissolved in deionized water at 85 °C under 600 rpm magnetic stirring for 3 h. Once fully solubilized and cooled to 25 °C, (AmB) (4 mg per 5 mL) was added and stirred gently until completely dispersed. To prevent the chemical degradation of (AmB) in the acidic chitosan/gelatin solution, a dual-nozzle electrospinning setup was employed—allowing the drug to be incorporated exclusively into the neutral PVA phase, while the acidic biopolymer blend was delivered separately. This strategy ensured drug stability and avoided denaturation caused by low pH conditions (Montenegro et al. 2020). Both solutions were stirred at 23 ± 2 °C for 24 h, followed by 1 h of degassing to eliminate entrapped air bubbles. The viscosity and conductivity of the polymer solutions were not quantitatively measured but were empirically optimized to ensure spinnability and fiber formation without bead defects.

### Dual-Nozzle electrospinning process

Electrospinning was carried out using a dual-nozzle horizontal electrospinning system (Fanavaran Nano-Meghyas, ESDP30, Iran), located at the Central Laboratory of the School of Advanced Technologies in Medicine, Tehran University of Medical Sciences. The setup included two horizontally aligned syringes (5 mL volume, 18G needles), each connected to independent digitally controlled syringe pumps. The syringes were loaded with equal volumes (5 mL each) of the chitosan/gelatin and PVA/(AmB) solutions, maintaining a 1:1 volumetric feed ratio throughout the process. A high-voltage power supply (35 kV max output) was used to apply a voltage of 20 kV, and the fibers were collected on a rotating stainless-steel cylindrical drum set at 162 rpm. The needle-to-collector distance was fixed at 120 mm, and each nozzle was translocated horizontally between 20 mm and 200 mm at 500 mm/min to ensure uniform coverage. The entire electrospinning process was conducted under ambient conditions (23 ± 2 °C, 35–40% relative humidity) and continued for approximately 10 h, resulting in a uniform nanofiber mat. The use of a dual-nozzle configuration allowed for random blending of the two polymer streams on the collector, forming a homogeneous AmB-loaded nanofiber structure without phase separation, as confirmed by SEM and FTIR analyses (see Results section). The final mats were deposited on aluminum foil substrates and stored in a desiccator to prevent moisture uptake before further characterization. All electrospinning experiments were performed in triplicate to ensure reproducibility and consistency across batches.

### Characterization of nanofibers

#### Scanning electron microscopy

The surface morphology of the electrospun nanofiber mats was examined using scanning electron microscopy (SEM) with a field-emission system (AIS2300C, SERON Technology, South Korea). Nanofiber samples (1 × 1 cm) were mounted onto aluminum stubs using double-sided carbon tape and sputter-coated with a ~ 10–15 nm gold layer (SCD 004, BAL-TEC, Switzerland). Imaging was performed under high-vacuum conditions (~ 10⁻⁶ Torr) at an accelerating voltage of 26.0 kV and a working distance of 8.1–8.3 mm in secondary electron imaging (SEI) mode. A high accelerating voltage of 26.0 kV was selected to enhance resolution without inducing thermal damage. Images were acquired at 25,000× magnification (scale bar: 1 μm). At least three independent nanofiber samples were imaged, and measurements were performed on multiple fields per sample. A total of 100 fibers were randomly selected and measured using calibrated ImageJ software (version 1.54 g, NIH, USA). Statistical outputs included mean, SD, minimum, maximum, and median values.

#### Fourier transform infrared spectroscopy

The chemical structures and functional groups of the individual polymers (chitosan, gelatin, and PVA), AmB, and the AmB-loaded electrospun nanofibers were analyzed using Fourier Transform Infrared (FTIR) spectroscopy in transmission mode (Tensor 27, Bruker, Germany). Nanofiber mats were ground into fine powder and mixed with dry potassium bromide (KBr) at a ratio of 1–2 mg sample to 100 mg KBr to prepare transparent pellets. Spectra were recorded over the wavenumber range of 4000–400 cm⁻¹ with a resolution of 4 cm⁻¹, averaging 32 scans per sample. Samples were dried under vacuum prior to analysis to eliminate moisture interference. Baseline correction and spectral processing were performed using OPUS software (Bruker, Germany), and air background spectra were collected before each scan. All FTIR measurements were performed in triplicate to ensure reproducibility.

#### X-ray diffraction analysis

The crystalline structure of PVA, gelatin, chitosan, AmB, and the AmB-loaded nanofiber mat was examined using X-ray diffraction (XRD) with an X’Pert Pro diffractometer (PANalytical, The Netherlands) equipped with Cu Kα radiation (λ = 1.5406 Å). Measurements were performed at 35 kV and 30 mA over a 2θ range of 5°–60°, with a step size of 0.02° and a scan time of 1 s per step. The slit configuration included a 1° divergence slit and a 0.3 mm receiving slit. Powdered forms of the individual components were prepared for analysis, while the AmB-loaded nanofiber mat was analyzed intact. All samples were evaluated in triplicate to ensure reproducibility of the diffraction patterns. Data processing was performed using X’Pert HighScore software.

#### Mechanical properties

The mechanical properties of the electrospun nanofiber mats were evaluated using a universal testing machine (Santam STM-20, Iran) equipped with a 200 N load cell. Nanofiber mats were cut into rectangular strips measuring 1 cm × 5 cm. Mechanical testing was conducted at a constant crosshead speed of 5 mm/min under controlled ambient conditions (temperature: 23 ± 2 °C; relative humidity: 50 ± 5%). From the resulting stress–strain curves, key mechanical parameters including tensile strength (MPa), elongation at break (%), elastic modulus (MPa), and energy to break (J) were calculated. Each experimental condition was tested in triplicate to ensure data consistency. Statistical analyses were performed using unpaired two-tailed t-tests, and differences were considered significant at p-values < 0.05.

### Drug loading and release

#### Drug content Estimation

To quantify the amount of (AmB) loaded into the nanofibers, square Sect. (2 × 2 cm) were randomly cut from different regions of the nanofiber mats. Samples were weighed using a high-precision analytical balance (Sartorius Entris^®^224i; readability: 0.1 mg). All measurements were performed in triplicate and reported as mean ± standard deviation (SD). The electrospinning solution was prepared by dissolving 10% (w/v) PVA in distilled water and blending it with 2% (w/v) gelatin and 1% (w/v) chitosan under continuous stirring at 60 °C for 4 h. (AmB) (10 mg) was then added to 5 mL of the blended polymer solution. After electrospinning and vacuum drying the fibers at 40 °C for 24 h, the theoretical drug content per unit area was calculated by dividing the total drug amount by the total area of the electrospun mat. Drug content per unit surface area (µg/cm²) was derived from the dimension equation based on:

$$\:\begin{array}{c}Drug\:Content\:(mg/cm^2)=Total\:Weight\:of\:Drug\\Used\:\left(mg\right)\:/Total\:Area\:of\:Nanofiber\:(cm^2)\end{array}\:$$

All measurements were conducted in triplicate to ensure reproducibility.

#### In vitro drug release assay

The release profile of (AmB) from the electrospun nanofibers was evaluated using the immersion method. A calibration curve was established using standard AmB solutions (0, 5, 10, 20, and 25 µg/mL), and absorbance was measured at 345 nm using a UV-Vis spectrophotometer (Lambda 25, Perkin Elmer, USA), based on the method of Nanda and Mishra (Nanda and Mishra 2011). Drug concentrations were quantified based on this previously established calibration curve. For the release study, 2 cm² samples of nanofiber mats were immersed in 20 mL of phosphate-buffered saline (PBS, pH 7.4) in 50 mL tightly sealed glass vials with screw caps to prevent evaporation. The vials were incubated at 37 ± 0.5 °C under static conditions. At predetermined time points (0, 1, 3, 5, 8, 10, 24, 48, and 72 h), the entire release medium was removed and replaced with an equal volume of fresh PBS maintained at the same temperature. This ensured consistent sink conditions throughout the experiment. All release experiments were performed in triplicate. Data were processed using Excel and GraphPad Prism v10.3.1 to plot cumulative release profiles over time. In addition, Fourier Transform Infrared (FTIR) spectroscopy was performed to qualitatively confirm the presence and structural integrity of AmB in the nanofiber matrix and to assess possible drug–polymer interactions, such as hydrogen bonding. FTIR data do not provide direct quantitative release values and thus do not directly correlate with UV–Vis measurements; rather, they serve as a complementary tool. Representative FTIR band positions and assignments for individual components and AmB-loaded composite nanofibers are provided in Table S2 of the Supplementary Material.

### Cell and parasite culture

#### THP-1 and HDF cells

Human monocytic leukemia (THP-1; ATCC TIB-202) and human dermal fibroblast (HDF) cells were obtained from the Pasteur Institute of Iran (Tehran, Iran). THP-1 cells were cultured in complete RPMI-1640 medium supplemented with 10% heat-inactivated fetal bovine serum (FBS), 1% penicillin-streptomycin, and 2 mM L-glutamine. HDF cells were maintained in Dulbecco’s Modified Eagle Medium (DMEM) with the same supplements. Both cell lines were incubated at 37 ± 0.5 °C in a humidified atmosphere containing 5% CO₂. THP-1 cells between passages 5–15 and HDF cells between passages 3–10 was used for all experiments to ensure biological consistency. Culture media for HDFs were refreshed every 2–3 days. All cell cultures were routinely tested and confirmed negative for mycoplasma contamination using standard culture-based detection methods according to established protocols (Uphoff and Drexler 2014).

#### Leishmania major promastigotes

Promastigotes of *L. major* (MRHO/IR/75/ER) strain were kindly obtained from the *Leishmania* Bank of the Leishmaniasis Laboratory, School of Public Health, Tehran University of Medical Sciences (Tehran, Iran). The parasites were cultured in sterile, upright T-25 tissue culture flasks (SPL Life Sciences, Korea) containing 5 mL of RPMI-1640 medium (Gibco, USA), supplemented with 10% heat-inactivated fetal bovine serum (FBS; Gibco, USA), 1% penicillin-streptomycin (100 IU/mL and 100 µg/mL, respectively), and 2 mM L-glutamine. Cultures were maintained at 25 ± 0.5 °C in a stationary incubator under standard atmospheric conditions (without CO₂) and sub-cultured every 4–6 days to sustain exponential and late logarithmic growth phases. For infection assays, promastigotes were harvested on day 7 post-subculture, corresponding to the stationary phase. Parasite concentration was adjusted using a Neubauer hemocytometer (Marienfeld, Germany) under light microscopy (400× magnification). The medium pH was regularly monitored and maintained between 6.8 and 7.2. All cultures were microscopically examined and confirmed to be axenic, with no bacterial or fungal contamination, prior to experimental use.

### In vitro biological assays

#### Cytotoxicity assay (THP-1)

The cytotoxicity of the nanofiber mats was evaluated using an indirect MTT assay based on ISO 10993-5 guidelines. Extracts of the nanofibers (6 cm², with 0%, 10%, and 20% drug-to-polymer weight ratios) were prepared according to ISO 10993-12 by incubating sterilized mats in complete RPMI-1640 medium at 37 °C for 24 h. THP-1 human monocytic cells were seeded in flat-bottom, tissue culture-treated 96-well plates (Corning, USA) at a density of 4 × 10³ cells/well and treated with 100 ng/mL phorbol 12-myristate 13-acetate (PMA; Sigma-Aldrich, USA) for 24 h to induce differentiation into macrophage-like cells. PMA was dissolved in DMSO and diluted in the culture medium to a final concentration of 100 ng/mL, with the final DMSO concentration kept below 0.1% (v/v). Following PMA treatment, cells were washed with phosphate-buffered saline (PBS) and incubated in fresh PMA-free medium for another 24 h to stabilize the phenotype. The culture medium was then replaced with the nanofiber extracts and incubated for an additional 24 h. Subsequently, 10 µL of MTT solution (0.5 mg/mL in PBS) was added to each well and incubated for 4 h at 37 °C. Formazan crystals were solubilized using 100 µL of analytical-grade DMSO (Merck, Germany), and absorbance was measured at 570 and 630 nm using a microplate reader (BioTek ELx800, USA). Cell viability was calculated relative to untreated control wells. All experiments were performed in triplicate.

#### Cytotoxicity assay (HDF)

Cells were seeded in flat-bottom, tissue culture-treated 96-well plates (Corning, USA) at a density of 1 × 10⁴ cells per well and cultured in Dulbecco’s Modified Eagle Medium (DMEM) supplemented with 10% heat-inactivated FBS, 1% penicillin-streptomycin, and 2 mM L-glutamine in a final volume of 200 µL per well. After a 24-hour incubation at 37 °C in a humidified 5% CO₂ atmosphere to allow adherence, cells were treated with (a) (AmB)-loaded nanofibers, (b) blank nanofibers, and (c) free (AmB) at various concentrations. After 24–48 h of incubation, 10 µL of MTT solution (5 mg/mL in PBS) was added to each well and incubated for 4 h. The supernatant was removed, and 100 µL of analytical-grade DMSO (Merck, Germany) was added to dissolve the formazan crystals. Absorbance was measured at 570 nm (reference 630 nm) using a microplate reader.

Cell viability was calculated using the formula:

$$\begin{array}{c}{Cell\:Viability\:\left(\%\right)=(OD\_sample\:-\:OD\_blank)\:}/\:\\\:(OD\_control\:-\:OD\_blank)\times\:\:100\end{array}\:$$

where OD (Optical Density) refers to the absorbance measured at 570 nm (with a reference wavelength of 630 nm), which reflects the amount of formazan produced by metabolically active cells.

Untreated cells served as negative controls, while cells treated with 200 µg/mL of AmB were used as positive cytotoxic controls. To assess the potency of this specific AmB preparation (the same used for loading into the nanofibers), IC₅₀ values were determined using serial dilutions of 1, 2.5, 5, 20, 100, and 200 µg/mL. All experiments were performed in triplicate and repeated independently three times. IC₅₀ values were determined by nonlinear regression using GraphPad Prism (version 10.3.1, GraphPad Software, San Diego, CA, USA).

#### Intracellular amastigote assay

Human monocytic THP-1 cells (ATCC TIB-202), obtained from the Pasteur Institute of Iran (Tehran, Iran), were cultured in RPMI-1640 medium supplemented with 10% heat-inactivated fetal bovine serum (FBS), 1% penicillin-streptomycin, and 2 mM L-glutamine. Cells were maintained in a humidified 5% CO₂ atmosphere at 37 ± 0.5 °C. For differentiation into macrophage-like cells, THP-1 cells were seeded in 8-well chamber slides (Nunc, Denmark) at a density of 1 × 10⁵ cells/well and treated with 100 ng/mL phorbol 12-myristate 13-acetate (PMA; Sigma-Aldrich, USA) for 24 h. The cells were then washed with PBS and incubated in PMA-free medium for an additional 24 h to stabilize the differentiated phenotype. *L. major* promastigotes (strain MRHO/IR/75/ER) were provided by the *Leishmania* Bank, School of Public Health, Tehran University of Medical Sciences. Parasites were cultured in RPMI-1640 medium supplemented with 10% FBS and 1% Pen/Strep at 25 °C and subcultured every 4–6 days. Promastigotes in the stationary growth phase (day 7 post-subculture) were counted using a Neubauer hemocytometer under light microscopy and adjusted to an appropriate density for infection. All cultures were routinely monitored and confirmed to be axenic and free from bacterial or fungal contamination. Differentiated THP-1 macrophages were infected with *L. major* promastigotes at a multiplicity of infection (MOI) of 5:1. After 18 h incubation at 37 °C, non-internalized parasites were removed by washing twice with antibiotic-free RPMI-1640 medium. The infected cells were then treated either with free (AmB) at concentrations of 200, 100, 20, 5, 2.5, and 1 µg/mL and with AmB-loaded nanofibers of different sizes (3 × 3 = 9 cm², 2 × 2 = 4 cm², 1 × 1 = 1 cm², 0.5 × 0.5 = 0.25 cm², and 0.25 × 0.25 = 0.0625 cm²), corresponding to drug contents of 163.62, 72.72, 18.18, 4.545, and 1.1362 µg, respectively. A total of 500 µL of each treatment was added per well, and plates were incubated for 48 h at 37 °C. Following treatment, chamber slides were air-dried, fixed with absolute methanol for 10 min, and stained with 1% Giemsa solution (Merck, Germany) for 30 min. The number of intracellular amastigotes in 100 macrophages per sample was counted under a light microscope at 1000× magnification. The survival index was calculated using the following equation, as previously described (Scariot et al. 2019): Survival Index = (Percentage of infected macrophages × Mean number of amastigotes per infected macrophage)/100.

### In vivo evaluation

#### Animal infection model

A total of 84 inbred male BALB/c mice (4–6 weeks old, 25–30 g) were obtained from the Pasteur Institute (Tehran, Iran). Mice were housed under standard laboratory conditions (12 h light/dark cycle, 40–50% humidity, 25 ± 3 °C) with free access to food and water. All animals were subcutaneously inoculated at the base of the tail with *L. major* (MRHO/IR/75/ER) promastigotes (2 × 10⁶ parasites in 0.2 mL PBS) using an insulin syringe under sterile conditions. Visible cutaneous lesions developed approximately 20 days post-infection. All animal procedures were conducted in accordance with the guidelines of the Institutional Animal Care and Use Committee (IACUC) and were approved by the Ethics Committee of the School of Public Health, Tehran University of Medical Sciences (TUMS), under protocol number IR.TUMS.AEC.1401.043.

#### Treatment protocol and grouping

Upon the development of visible cutaneous lesions (~ 20 days post-inoculation), 84 BALB/c mice were randomly divided into seven experimental groups (*n* = 12 per group), each receiving a specific treatment protocol as summarized in (Table 1). Treatments varied in terms of formulation, route of administration, frequency, and total duration.

**Table 1:** Experimental groups, treatment protocols, and observation periods

Group	Treatment	Formulation	Route of Administration	Frequency	Duration	Observation Period
1	Glucantime^®^ (Positive Control)	100 mg/kg Glucantime^®^	Intraperitoneal injection (IP)	Daily	14 days	21 days
2	SinaAmpholeish^®^ (Topical)	0.4% Liposomal (AmB) ointment	Topical	Every 12 h	56 days	63 days
3	AmB-loaded nanofiber patch (biweekly)	Chitosan/Gelatin/PVA + AmB	Topical patch	Every other day	14 days	21 days
4	AmB-loaded nanofiber patch (weekly)	Chitosan/Gelatin/PVA + AmB	Topical patch	Weekly	56 days	63 days
5	Blank nanofiber patch (biweekly)	Chitosan/Gelatin/PVA (no drug)	Topical patch	Every other day	14 days	21 days
6	Blank nanofiber patch (weekly)	Chitosan/Gelatin/PVA (no drug)	Topical patch	Weekly	56 days	63 days
7	Untreated control (Negative Control)	None	None	None	None	63 days

To reduce animal use while ensuring statistical validity, Groups 2 and 7 were included in both short-term (21-day) and long-term (63-day) comparisons. Nanofiber patches were cut to exceed lesion margins slightly and fixed in place using sterile medical adhesive (Angio-catheter roll). Patches were replaced according to the assigned schedule. All treatments and lesion size measurements were conducted by a single trained investigator to minimize inter-operator variability. Mice exhibiting signs of distress (e.g., excessive lesion size, tremors, lethargy, or anorexia) were euthanized in accordance with ethical institutional guidelines. Any resulting missing data were treated as Missing Completely at Random (MCAR) during statistical analysis.

#### Lesion size measurement

Lesion size was assessed weekly using a digital Vernier caliper (accuracy ± 0.01 mm) in two perpendicular directions (length and width). The first measurement was performed prior to the initiation of treatment, and subsequent measurements were repeated on a weekly basis. In all groups, lesion size was also measured one week after the end of the respective treatment protocol to monitor any delayed effects. For each lesion, the average of two perpendicular measurements was recorded. Wound area (A) was calculated using the following formula: A = (length × width)/2.

#### Parasite load assessment

At the study start and endpoint, impression smears were prepared from lesion margins of two mice of each group. The smears were air-dried, fixed with absolute methanol, and stained with Giemsa stain (Merck, Germany). Amastigote counts were conducted under a light microscope (Carl Zeiss, Germany) at 1000× magnification. Parasite load was semi-quantitatively graded following WHO guidelines (World Health Organization 1990):

- ++++ >10 amastigotes per microscopic field.
- +++ 1–10 amastigotes per field.
- ++ 11–100 amastigotes per 100 fields.
- + 1–10 amastigotes per 100 fields.
-: 0 amastigote per 1000 field.

All slides were blindly evaluated by two independent examiners. Results were reported as the average of both readings per sample. Animal procedures complied with the Institutional Animal Care and Use Committee (IACUC) guidelines and were approved by the Ethics Committee of the School of Public Health, Tehran University of Medical Sciences (TUMS) under protocol number IR.TUMS.AEC.1401.043.

### Statistical analysis

All statistical analyses were performed using GraphPad Prism version 9.0.0. For descriptive statistics, data were reported as mean ± standard deviation (SD), with additional parameters such as minimum, maximum, and range calculated when applicable. Normality was assessed using the Shapiro–Wilk test, and equality of group variances was evaluated using Levene’s test. Independent Student’s t-test was used to compare fiber diameters between blank and drug-loaded samples, while nonlinear regression based on the four-parameter logistic (4PL) model was applied to determine IC₅₀ values from cytotoxicity assays. For lesion size comparisons, one-way ANOVA with Tukey’s post hoc test (for parametric data) and Mann–Whitney U test (for non-parametric data) were used. A p-value < 0.05 was considered statistically significant.

## Results

### Nanofiber fabrication and characterization

#### Morphology and diameter distribution

**Fig. 1:**
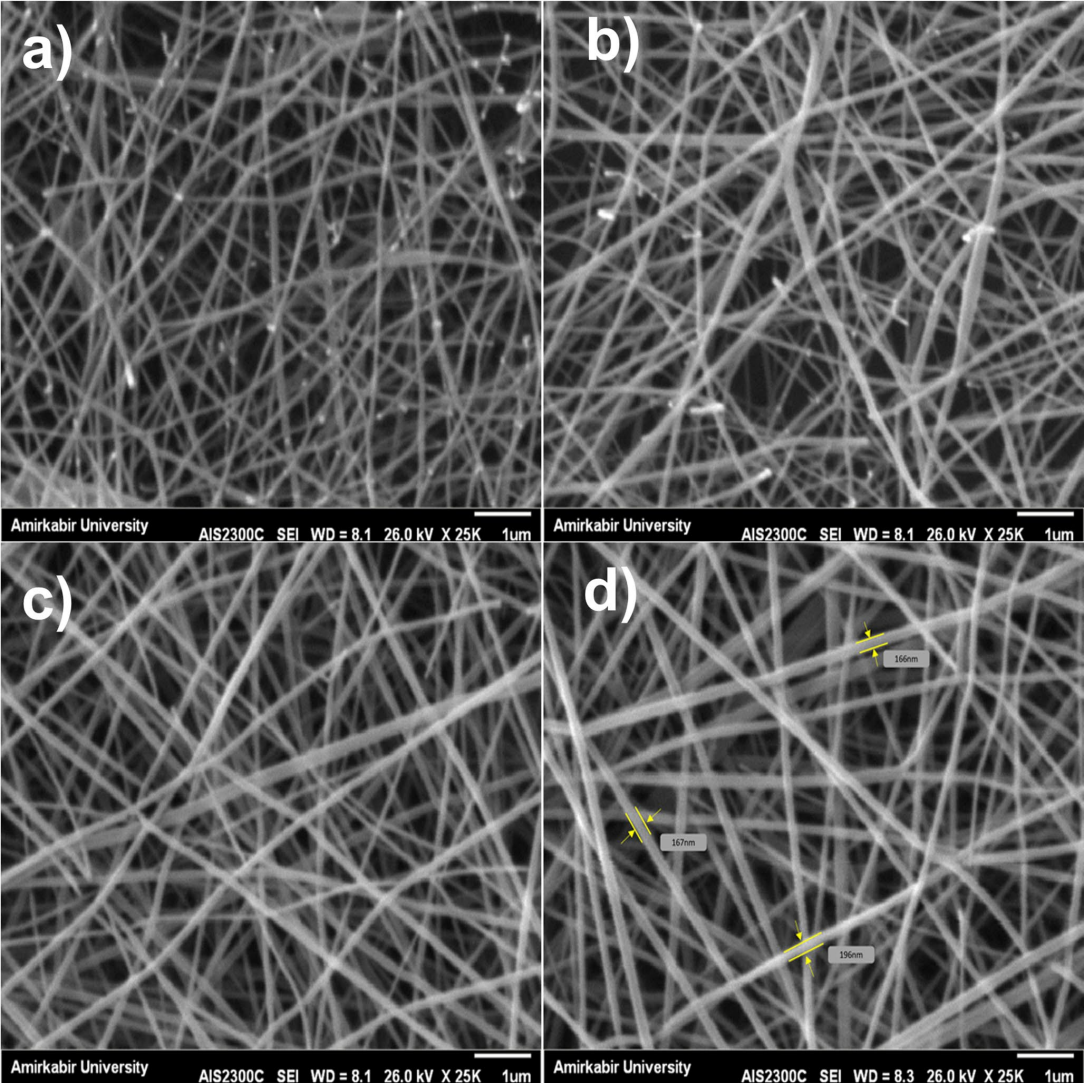
SEM micrographs of electrospun chitosan/gelatin/PVA/AmB nanofibers. (**a**) Overall fiber morphology at 25,000× magnification. (**b**) Enlarged view showing smooth, bead-free surface. (**c**) Random fiber orientation across the mat. (**d**) Annotated fiber diameters demonstrating consistent dimensions. All images were acquired using the AIS2300C SEM system under SEI mode at 26.0 kV and 8.1–8.3 mm working distance

**Fig. 2:**
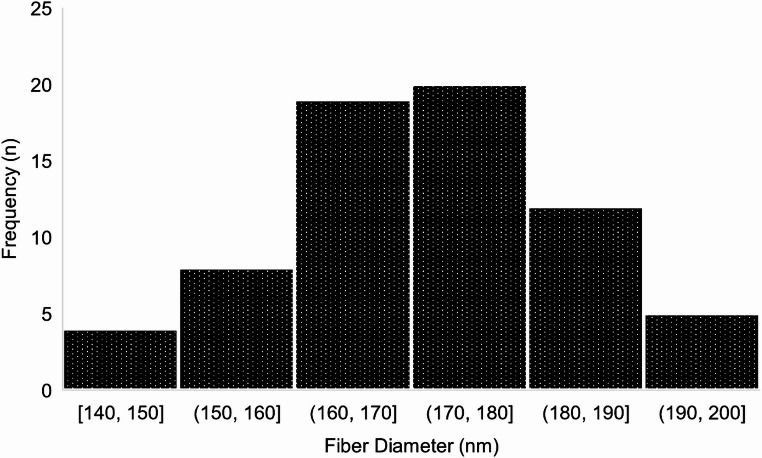
Histogram showing the distribution of fiber diameters in electrospun nanofibers. The majority of fibers fall within the 160–180 nm range, indicating a relatively uniform diameter with slight variation across the sample

SEM imaging confirmed the successful fabrication of electrospun chitosan/gelatin/PVA/AmB nanofibers exhibiting uniform morphology and smooth, bead-free surfaces. The nanofibers displayed consistent structural characteristics, with random orientation and no signs of bead formation, suggesting a stable and reproducible electrospinning process. Representative SEM micrographs are presented in (Fig. 1). (Fig. 1A) displays the overall morphology at 25,000× magnification. (Fig. 1B) shows an enlarged view of the nanofiber surface, while (Fig. 1C) illustrates the random fiber orientation across the mat. Representative diameter measurements ranging from 166 to 196 nm are annotated in (Fig. 1D). Quantitative analysis based on 100 measurements revealed a mean fiber diameter of 176 ± 15 nm, with a minimum of 138 nm and a maximum of 205 nm. The corresponding histogram (Fig. 2) displayed a near-Gaussian distribution, with the majority of fibers falling within the 150–200 nm range. No statistically significant difference (*p* > 0.05) in fiber diameters was observed between blank and (AmB)-loaded nanofibers, as determined by an unpaired Student’s t-test. All SEM images were acquired at ambient conditions (23 ± 2 °C, 30–40% RH) using the AIS2300C SEM system. The fiber diameters were highly consistent, indicating good control over the electrospinning process and confirming the reproducibility of fiber morphology.

#### Chemical characterization by FTIR

**Fig. 3:**
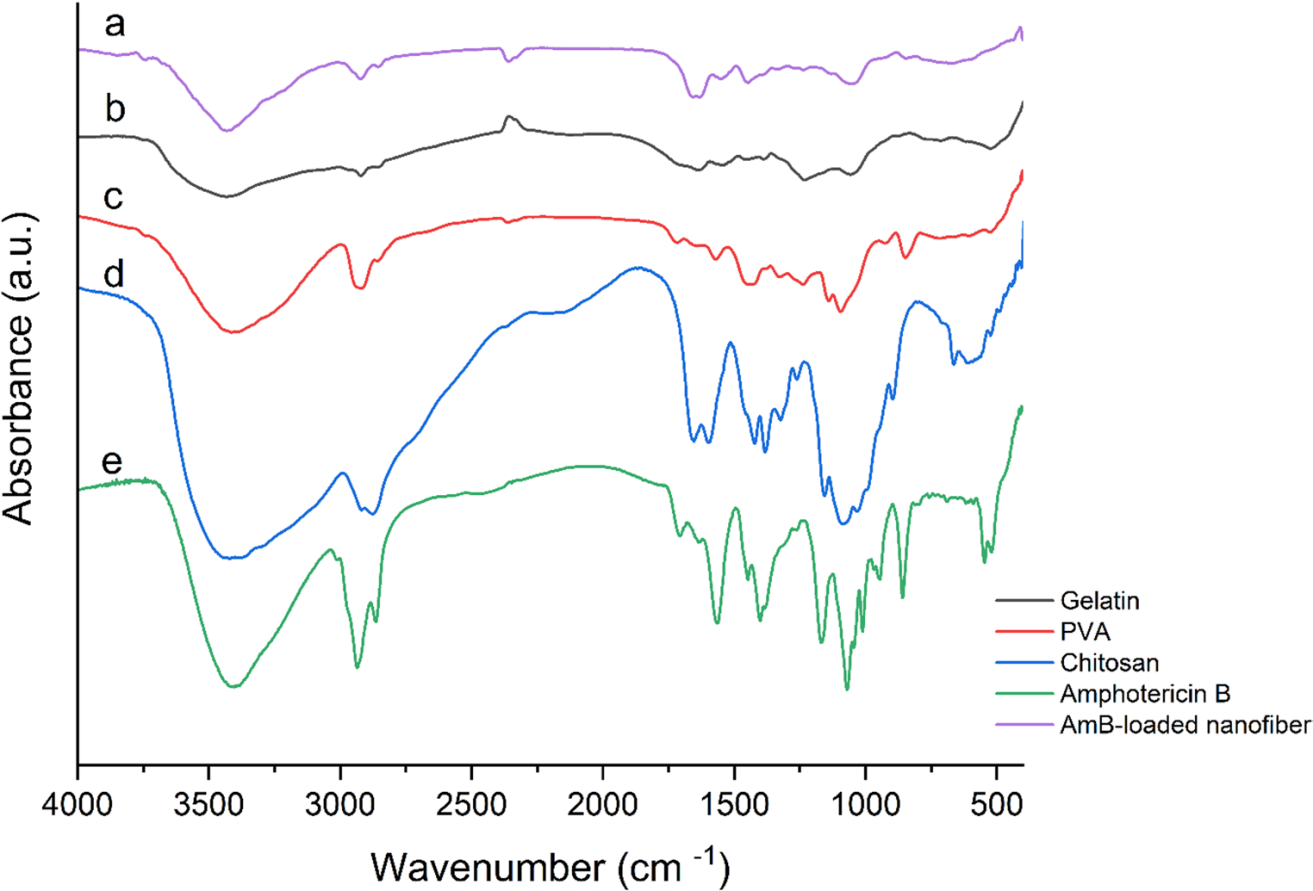
Normalized and baseline corrected FTIR spectra of the individual components and the final AmB-loaded nanofibers. Spectra are shown for gelatin (black), PVA (red), chitosan (blue), (AmB) (green), and the AmB-loaded chitosan/gelatin/PVA nanofibers (purple). The AmB-loaded fiber spectrum exhibits a combination of major peaks from all components. Broadening and slight shifts of key bands—particularly near 3300 cm⁻¹—suggest hydrogen bonding and physical entrapment of (AmB) within the polymeric nanofiber matrix, confirming successful drug incorporation and chemical compatibility among constituents

The FTIR spectra of individual components including polyvinyl alcohol (PVA), gelatin, chitosan, and (AmB), along with the final AmB-loaded nanofibers, are shown in (Fig. 3). All spectra were normalized and baseline-corrected to facilitate comparative analysis. PVA exhibited characteristic peaks at ~ 3280–3300 cm⁻¹ (O–H stretching), ~ 2910 cm⁻¹ (C–H stretching), and a strong band at ~ 1140 cm⁻¹ corresponding to C–O–C stretching. A peak at ~ 840 cm⁻¹ was also observed (C–C skeletal vibrations). Gelatin displayed typical amide bands at ~ 3300–3400 cm⁻¹ (O–H and N–H stretching), ~ 1640 cm⁻¹ (amide I), ~ 1540 cm⁻¹ (amide II), and ~ 1240 cm⁻¹ (amide III). Chitosan exhibited bands near ~ 3350 cm⁻¹ (O–H/N–H), ~ 1650 cm⁻¹ (amide I), ~ 1560 cm⁻¹ (amide II), and ~ 1020–1150 cm⁻¹ (C–O stretching). (AmB) showed peaks near ~ 3400 cm⁻¹ (O–H stretching), ~ 1700 cm⁻¹ (C = O), 1450–1600 cm⁻¹ (C = C polyene backbone), and 1100–1200 cm⁻¹ (C–O). The FTIR spectrum of the final AmB-loaded nanofibers spectrum a in (Fig. 3) revealed a combination of all major peaks from the constituent components, confirming their successful incorporation. Broadening and slight shifts of key absorption bands, particularly near 3300 cm⁻¹, indicate hydrogen bonding and physical entrapment of (AmB) within the nanofiber matrix. As presented in (Table 2), a comparative summary of major absorption bands helps validate the molecular interactions and structural compatibility between the components in the AmB-loaded formulation.

**Table 2:** Comparative summary of key FTIR absorption bands for individual components and the composite nanofiber

Material	Key Peaks (cm⁻¹)	Functional Groups
PVA	3280–3300, 2910, 1140, 840	O–H, C–H, C–O–C, C–C
Gelatin	3300–3400, 1640, 1540, 1240	Amide A, I, II, III
Chitosan	3350, 1650, 1560, 1020–1150	O–H/N–H, Amide I/II, C–O stretching
(AmB)	3400, 1700, 1450–1600, 1100–1200	O–H, C = O, C = C, C–O
AmB-loaded nanofiber	3300, 1650, 1540, 1140	Overlapping bands, hydrogen bonding

#### X-ray diffraction

**Fig. 4:**
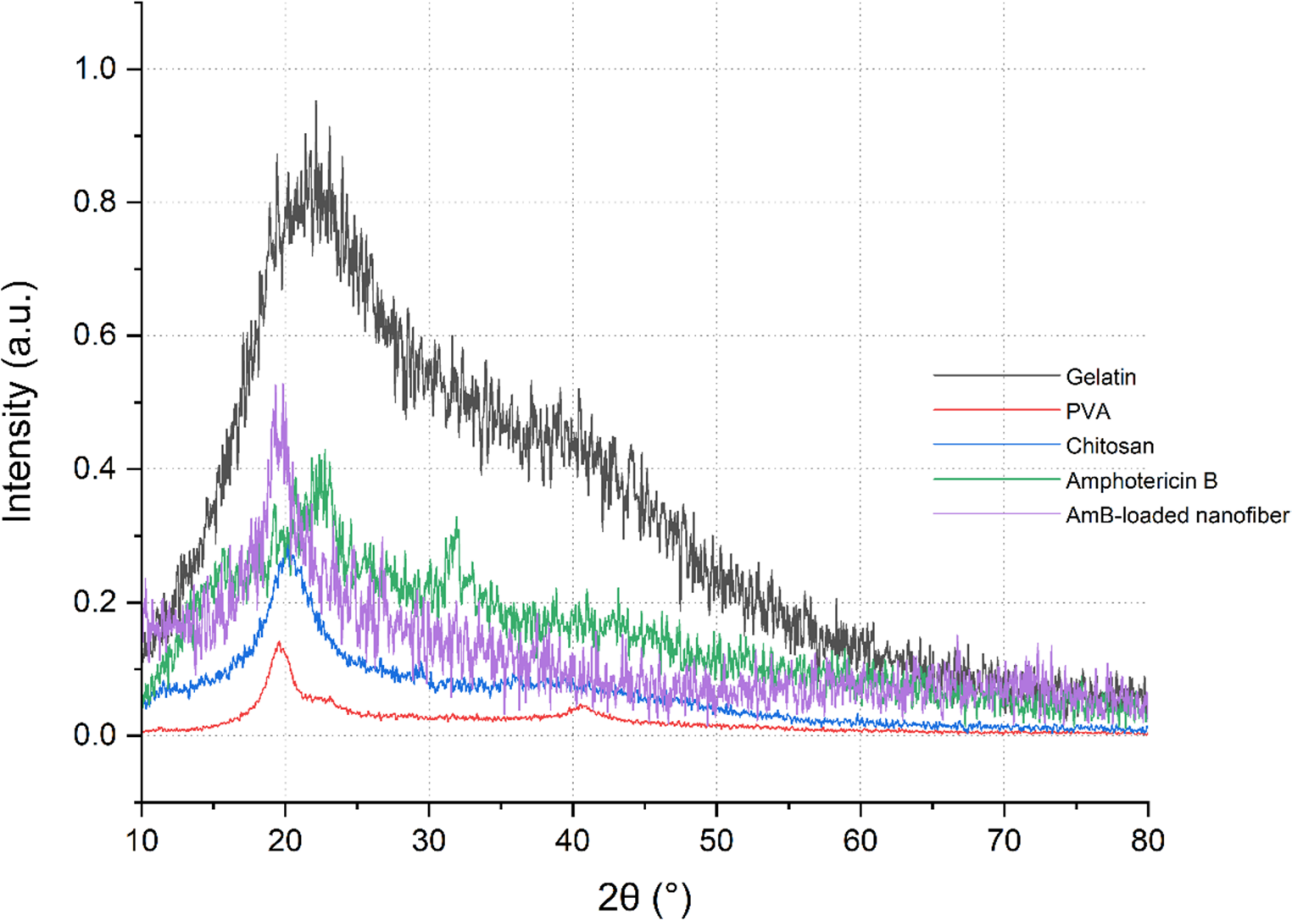
X-ray diffraction (XRD) patterns of individual components (gelatin, PVA, chitosan, and (AmB)) and the final AmB-loaded nanofiber. Gelatin and (AmB) display broad amorphous halos, while PVA exhibits sharp peaks at 2θ ≈ 20° and 41°, indicative of its semi-crystalline structure. Chitosan shows a broad peak at 2θ ≈ 20°, consistent with partial crystallinity. The AmB-loaded nanofiber spectrum exhibits a broadened peak at 2θ ≈ 20°, reflecting the overlap of polymeric contributions and confirming reduced crystallinity due to the incorporation of amorphous constituents

The XRD profile of the final AmB-loaded nanofiber (Fig. 4) revealed a significant reduction in crystallinity, as evidenced by the absence of sharp peaks and the presence of a broad halo near 2θ ≈ 20°. This can be attributed to the incorporation of amorphous constituents such as gelatin and (AmB), as well as the disruption of PVA’s crystalline domains by polymer blending. The observed loss of crystallinity is consistent with the literature and supports the enhanced water uptake, matrix swelling, and sustained drug release behavior of the electrospun scaffold, as summarized in (Table 3). Similar XRD profiles for PVA-based electrospun systems exhibiting reduced crystallinity upon incorporation of amorphous additives have also been reported in previous studies (Blazek and Copeland 2008; Kristensen et al. 2021).

**Table 3:** Summary of key XRD diffraction peaks and corresponding structural interpretations

Material	Major Peaks (2θ)	Structural Interpretation
PVA	20°, 41°	Semi-crystalline structure
Chitosan	20° (broad)	Partially crystalline
Gelatin	Broad halo ~ 20°	Amorphous
(AmB)	Broad halo ~ 20°	Amorphous
AmB-loaded nanofiber	Broad peak ~ 20°	Reduced crystallinity due to amorphous content

#### Mechanical properties

Tensile testing of the electrospun chitosan/gelatin/PVA nanofiber mats revealed favorable mechanical properties suitable for biomedical applications. The nanofibers exhibited a mean tensile strength of 1.66 ± 2.49 MPa, indicating their ability to withstand uniaxial stress without compromising integrity. The elongation at break was measured at approximately 1.34%, reflecting moderate flexibility, while the elastic modulus was calculated as 230.17 ± 177.64 MPa, demonstrating an acceptable level of stiffness and resistance to deformation. The energy to break, calculated from the area under the stress–strain curve, was found to be 0.95 J, confirming that the nanofiber mats are capable of absorbing mechanical load prior to failure. These values are consistent with those reported for other blended polymeric nanofibers intended for skin-contact or wound-healing applications and suggest that the fabricated mats possess sufficient mechanical resilience for handling, application, and conformability to irregular wound surfaces.

#### Drug encapsulation and loading efficiency

Following fabrication, approximately 550 cm² of nanofiber mat was obtained with a total dry weight of 361.2 mg. Based on the initial incorporation of 10 mg of (AmB), the theoretical drug loading was calculated as the average drug content per unit area was 18.18 ± 0.12 µg/cm². These values confirm the successful incorporation of (AmB) into the nanofiber matrix. For practical estimation, a 4 cm² section was expected to contain approximately 72.72 µg of the drug, a 1 cm² Sect. 18.18 µg, while smaller segments such as 0.25 cm² and 0.0625 cm² were estimated to contain 4.55 µg and 1.14 µg, respectively. This uniform distribution supports the reproducibility of the electrospinning process and highlights the potential of the fabricated fibers for localized and controlled drug delivery applications.

### In vitro drug release profile

#### Release trends over time

**Fig. 5:**
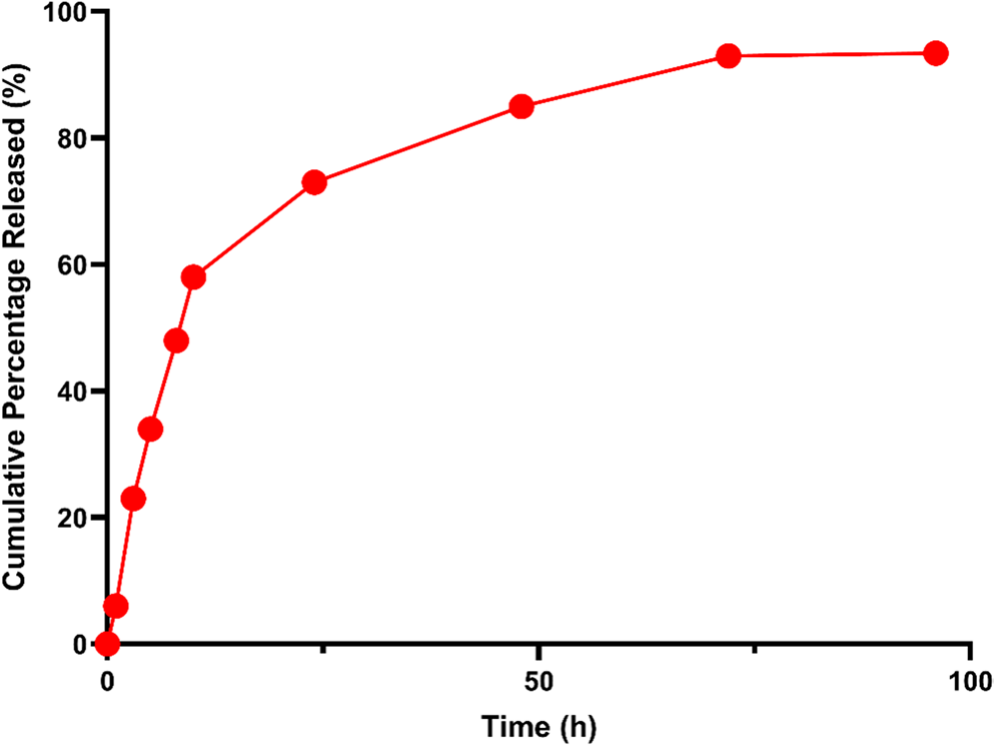
Time-dependent cumulative in vitro release profile of (AmB) from electrospun chitosan/gelatin/PVA nanofibers over a 96-hour period. The release study was performed under conditions simulating leishmanial wound environment (pH 6.7, 37 °C), in line with reported physiological pH ranges of 5.5–6.7 in cutaneous leishmaniasis lesions. Drug release was quantified at predefined intervals using spectrophotometric analysis. A rapid initial burst release was observed during the first 10 h, followed by a sustained and gradual release phase, reaching approximately 93.4% cumulative release at 96 h. These results indicate a biphasic release pattern suitable for localized and prolonged antileishmanial therapy

The in vitro drug release profile of the electrospun nanofiber formulation was assessed over a 96-hour period using 2 × 2 cm fiber patches. Drug release was quantified using a UV-Vis spectrophotometer at 345 nm at predefined intervals (0, 1, 3, 5, 8, 10, 24, 48, 72, and 96 h), with all measurements performed in triplicate. The cumulative percentage of released drug was calculated based on a standard calibration curve, referencing the total initial drug loading (72.72 µg). The nanofiber formulation exhibited a biphasic release pattern, with a rapid initial release phase during the first 10 h, reaching approximately 58% of the total drug, likely due to surface-associated drug. This was followed by a sustained and controlled release phase, culminating in ~ 93.4% cumulative release at 96 h. This biphasic behavior is characteristic of diffusion-mediated drug delivery systems, where an initial burst is followed by gradual release from within the matrix, supporting the potential of the formulation for long-acting topical antileishmanial therapy. The cumulative release data are graphically illustrated in (Fig. 5).

The release study was performed under conditions simulating leishmanial wound environment (pH 6.7, 37 °C), in line with reported physiological pH ranges of 5.5–6.7 in cutaneous leishmaniasis lesions. Drug release was quantified at predefined intervals using spectrophotometric analysis. A rapid initial burst release was observed during the first 10 h, followed by a sustained and gradual release phase, reaching approximately 93.4% cumulative release at 96 h. These findings demonstrate a biphasic release pattern, supporting localized and prolonged antileishmanial therapy.

### In vitro biological evaluation

#### THP-1 cell viability

**Fig. 6:**
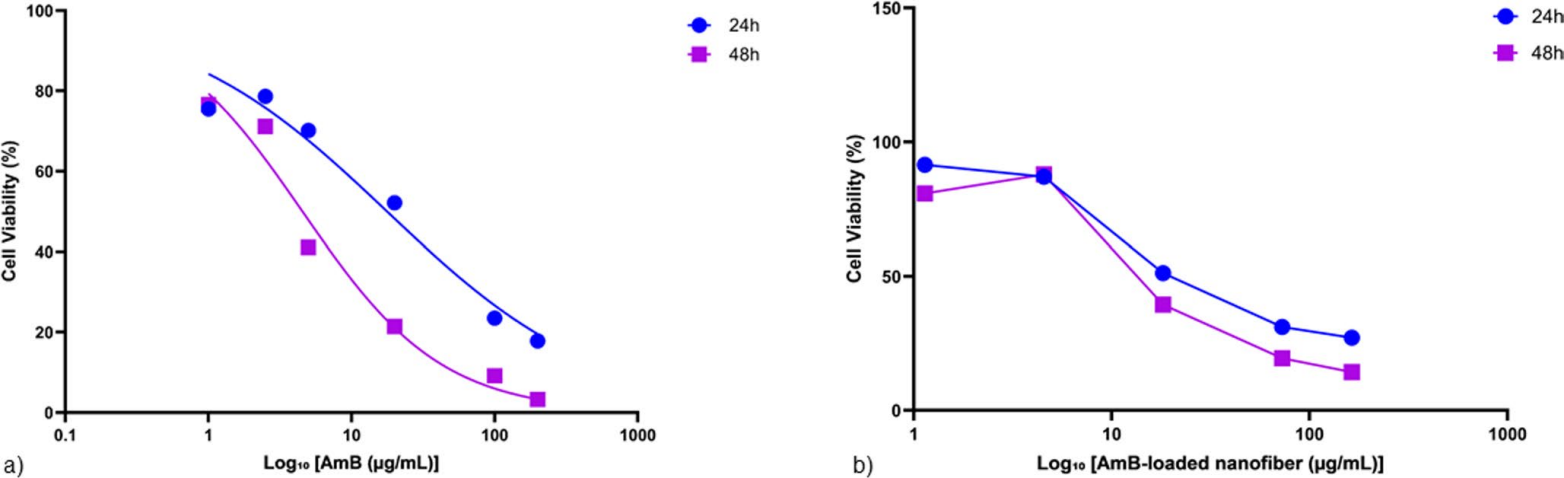
Dose–response curves showing the cytotoxicity of amphotericin B (AmB) and AmB-loaded nanofibers on THP-1 cells after 24 h (blue) and 48 h (purple) of exposure, as assessed by the MTT assay. (**a**) Cells treated with free AmB (1–200 µg/mL) exhibited IC₅₀ values of 17.73 ± 4.44 µg/mL at 24 h and 4.54 ± 1.06 µg/mL at 48 h, indicating increased cytotoxicity with longer exposure. (**b**) Treatment with AmB-loaded nanofibers (equivalent to 1–163 µg/mL AmB) resulted in IC₅₀ values of 28.38 ± 7.23 µg/mL (24 h) and 15.65 ± 3.22 µg/mL (48 h), reflecting a more gradual cytotoxic response compared to free AmB

The cytotoxic effects of free AmB and AmB-loaded nanofibers on THP-1 cells were evaluated using the MTT assay after 24 and 48 h of exposure. All experiments were performed in triplicate, and cell viability was normalized to untreated control cells (100% viability). As shown in (Fig. 6a), free AmB exhibited significant, time-dependent cytotoxicity. Nonlinear regression revealed that the IC₅₀ decreased markedly from 17.73 ± 4.44 µg/mL at 24 h to 4.54 ± 1.06 µg/mL at 48 h, reflecting enhanced cytotoxicity with prolonged exposure. In contrast, (Fig. 6b) illustrates the viability curves for THP-1 cells treated with AmB-loaded nanofiber. A more gradual decline in cell viability was observed for the nanofiber formulation. The IC₅₀ values were notably higher than the free drug, measured at 28.38 ± 7.23 µg/mL (24 h) and 15.65 ± 3.22 µg/mL (48 h), indicating a slower onset of cytotoxicity likely due to the sustained drug release from the nanofiber matrix. Together, these findings demonstrate that encapsulation of AmB in nanofibers attenuates its acute cytotoxic effects, especially within the first 24 h. This delayed response corresponds with the in vitro release profile of AmB from the nanofibers, where approximately 73% of the drug is released within the first 24 h, followed by only 12% during the second 24-hour period. These findings demonstrate that nanofiber encapsulation of AmB significantly increases cellular tolerance and reduces toxicity toward THP-1 cells compared to the free drug, which may be attributed to the controlled and sustained release profile of AmB from the nanofiber matrix. Furthermore, the gradual release of AmB appears to provide sufficient time for THP-1 cells to activate protective pathways and repair mechanisms, thereby reducing drug-induced cellular damage.

#### HDF cell viability

**Fig. 7:**
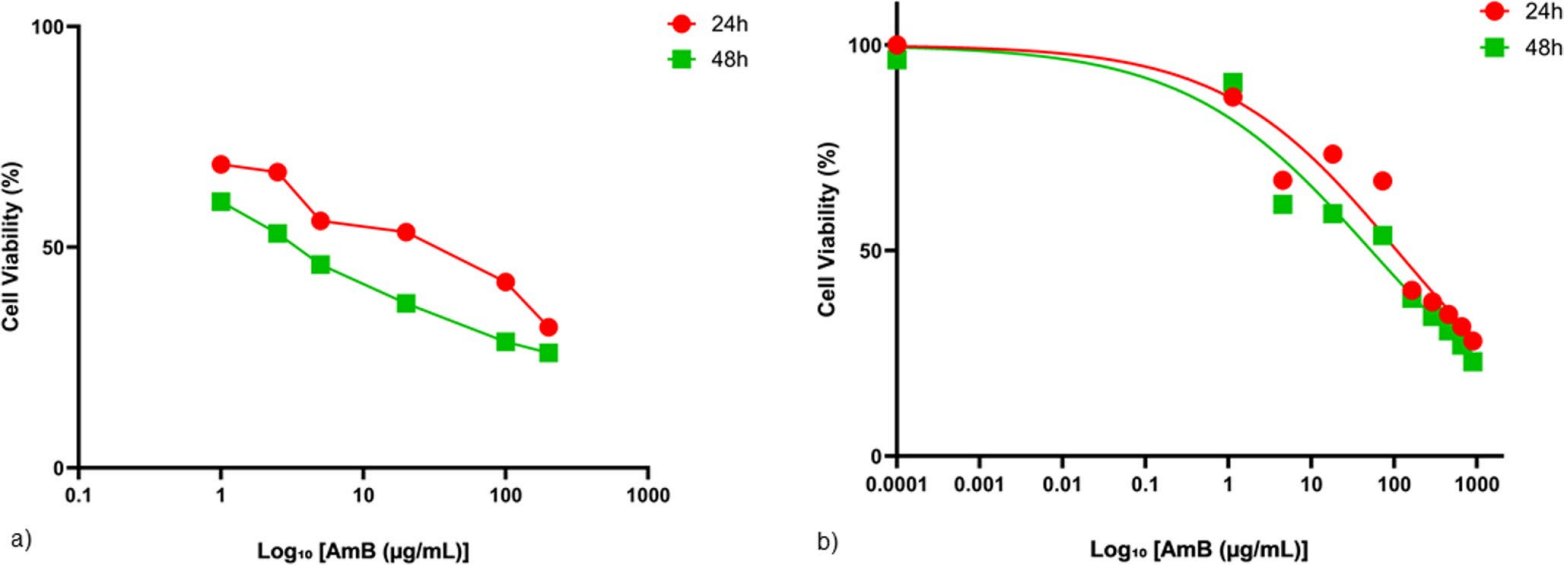
Cytotoxicity profiles of free amphotericin B (AmB) and AmB-loaded nanofibers against human dermal fibroblast (HDF) cells after 24 h (red) and 48 h (green) of exposure, as determined by the MTT assay. (**a**) Free AmB (1–200 µg/mL) exhibited IC₅₀ values of 21.58 ± 6.45 µg/mL (24 h) and 3.67 ± 0.78 µg/mL (48 h), with R² = 0.9830 and 0.9929, respectively. (**b**) AmB-loaded nanofibers (equivalent to 1–163 µg/mL AmB) showed IC₅₀ values of 107.9 ± 35.19 µg/mL (24 h) and 52.83 ± 15.51 µg/mL (48 h), with R² = 0.9367 and 0.9561, respectively, indicating markedly lower cytotoxicity compared with free AmB

The cytotoxic effects of both free AmB and AmB-loaded nanofibers were evaluated on human dermal fibroblast (HDF) cells using the MTT assay after 24 and 48 h of exposure. A broad range of concentrations was tested for each formulation to assess dose-dependent effects on cell viability. As shown in (Fig. 7a), free AmB exhibited marked cytotoxicity with a sharp reduction in viability, particularly at higher concentrations and longer exposure durations. Nonlinear regression analysis revealed a significant (*p* < 0.05, considered statistically significant as specified in the Materials and Methods section) decrease in IC₅₀ from 21.58 ± 6.45 µg/mL at 24 h to 3.672 ± 0.779 µg/mL at 48 h, indicating a clear time-dependent increase in cytotoxicity. The model demonstrated excellent goodness of fit, with R² values of 0.9830 and 0.9929, respectively. In contrast, the nanofiber-based formulation displayed a more gradual cytotoxic profile (Fig. 7b), consistent with sustained drug release from the polymeric matrix. The IC₅₀ values for AmB-loaded nanofiber were substantially higher than those of the free drug, measured at 107.9 ± 35.2 µg/mL (24 h) and 52.83 ± 15.51 µg/mL (48 h), with corresponding R² values of 0.9367 and 0.9561. Notably, AmB-loaded nanofiber-maintained cell viability above 60% at concentrations ≤ 5 µg/mL, whereas free AmB reduced viability below 40% under similar conditions. Collectively, these findings indicate that nanofiber-mediated delivery of AmB significantly attenuates its acute cytotoxicity toward HDF cells, especially during the initial 24 h of exposure. This protective effect is likely attributed to the diffusion-controlled release of AmB from the nanofibrous scaffold, which delays peak drug concentration at the cellular interface. In addition, the slower release of AmB from the nanofibers appears to provide sufficient time for normal cells to adapt and repair drug-induced damage, further contributing to improved cell viability. The substantial differences in IC₅₀ values between the two formulations highlight the potential of AmB-loaded nanofiber as a safer alternative for localized cutaneous leishmaniasis therapy.

#### Intracellular amastigote assay

**Fig. 8:**
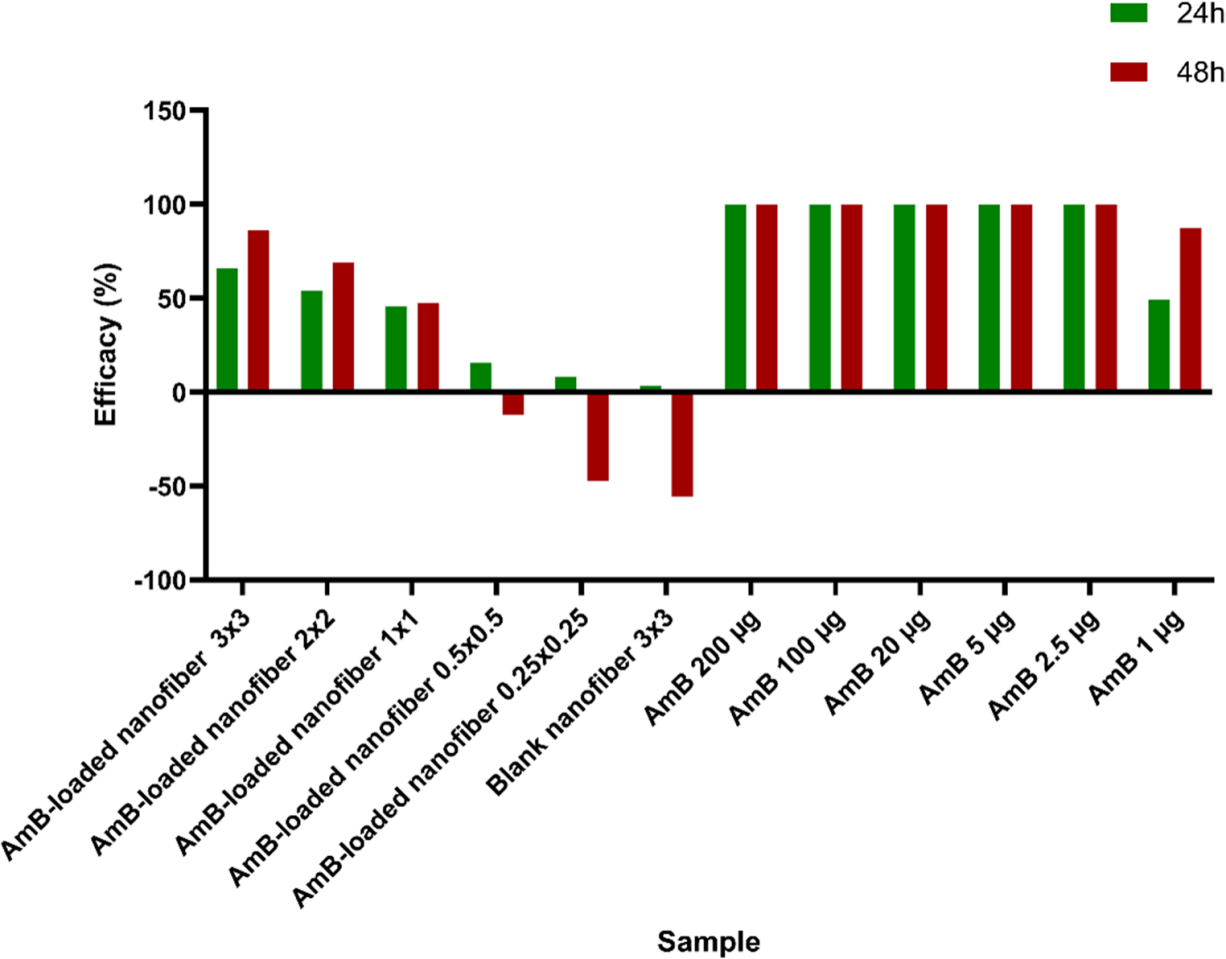
Antileishmanial activity of AmB and AmB-loaded nanofibers at 24 h (green) and 48 h (red). Bar graph shows the percentage inhibition of intracellular *L. major* amastigotes in THP-1 cells. Free AmB at all tested concentrations (5–200 µg/mL) resulted in complete parasite clearance (100%). Nanofiber patches of different sizes (3 × 3, 2 × 2, 1 × 1, 0.5 × 0.5, and 0.25 × 0.25 cm²), corresponding to drug contents of 163.62, 72.72, 18.18, 4.545, and 1.1362 µg AmB, respectively, showed a size-dependent antileishmanial effect, with the largest patches exhibiting the highest efficacy

As illustrated in (Fig. 8), AmB-loaded nanofibers demonstrated a size- and time-dependent antileishmanial activity against intracellular *L. major* amastigotes in THP-1 macrophages. Larger fiber patches (3 × 3 and 2 × 2 cm²) showed notable efficacy, with maximum parasite inhibition reaching 66.03% and 54% at 24 h, which increased to 86% and 69% at 48 h, respectively. Medium-sized fibers (1 × 1 cm²) provided moderate effects, while smaller patches (≤ 0.5 × 0.5 cm²) exhibited minimal or negative efficacy, including negative values at 48 h (e.g., − 12% and − 19%), indicating possible parasite proliferation due to subtherapeutic drug release.

All doses of free AmB (≥ 2.5 µg) achieved complete eradication of intracellular parasites at both time points (100% efficacy), confirming its potent leishmanicidal activity. AmB 1 µg showed lower efficacy at 24 h (49%) but achieved 87% efficacy at 48 h. Interestingly, blank (drug-free) nanofibers induced a slight transient reduction in parasite burden at 24 h (3.2%), which turned negative by 48 h (− 23%), suggesting that the physical presence of the nanofiber scaffold alone is insufficient for sustained antiparasitic activity. These findings highlight the necessity of sufficient drug content and optimal nanofiber surface area to ensure effective intracellular delivery and sustained therapeutic outcomes.

### In vivo therapeutic evaluation

#### Short-term lesion progression

##### Short-term lesion progression (Days 0–21)

**Fig. 9:**
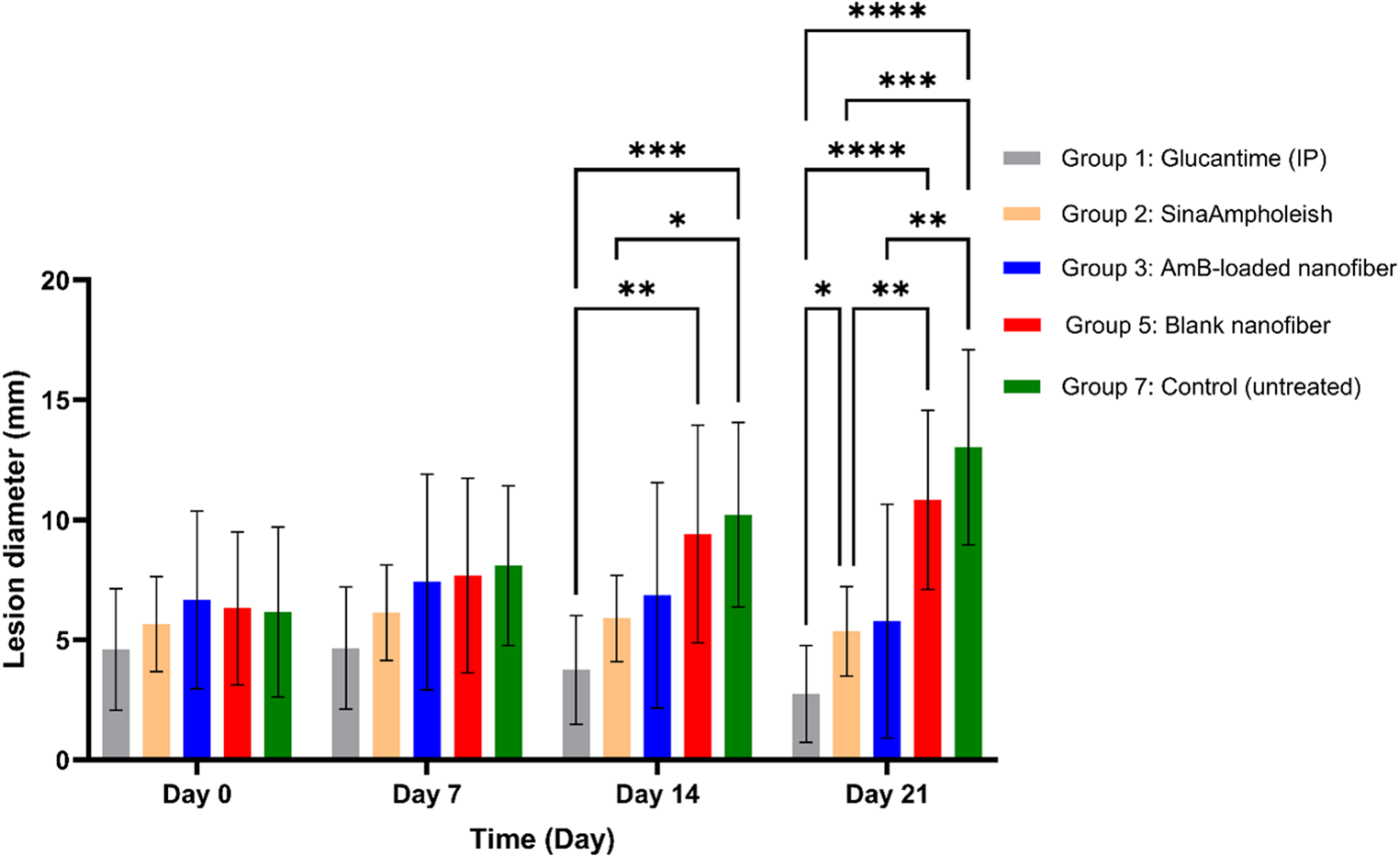
Representative lesion size from short-term treatment groups (Groups 1, 2, 3, 5, and 7) on Days 0, 7, 14, and 21. Mice treated with intraperitoneal Glucantime^®^ (Group 1) exhibited the most visible lesion size reduction. Lesions in the AmB-loaded nanofiber group (Group 3) also regressed significantly, showing comparable visual improvement to the SinaAmpholeish^®^ group (Group 2). In contrast, blank nanofibers and the untreated control groups showed progressive lesion enlargement

The progression of lesion size in short-term treatment groups was evaluated on Days 0, 14, and 21. Comparative results revealed that intraperitoneal Glucantime^®^ (Group 1) achieved the most substantial reduction, followed by AmB-loaded nanofibers (Group 3) and SinaAmpholeish^®^ (Group 2), with no significant difference observed between Groups 2 and 3. In contrast, lesion size increased in the untreated control and blank nanofiber groups. Detailed statistical comparisons for each group and timepoint are summarized in (Table 4), which illustrates the therapeutic performance during the early intervention phase. The visual appearance of lesions in representative mice from each group is further shown in (Fig. 9), highlighting the reduction or progression of lesions over the first 21 days.

**Table 4:** Lesion size comparison table (Days 0–21)

Day	Group	Outcome	*P*-value
Day 0	All Groups	No significant differences in baseline lesion size; indicates randomization and homogeneity.	*P* > 0.05
Day 14	Group 1 (Glucantime^®^)	Greater lesion size reduction vs. Group 5 and Group 7. ⁽¹⁾	*P* = 0.01 vs. G5; *P* = 0.0008 vs. G7
Day 14	Group 2 (SinaAmpholeish^®^)	Significant lesion reduction vs. control.	*P* = 0.0211 vs. G7
Day 14	Group 2 vs. Group 3	No significant difference observed; similar therapeutic outcomes.	NS )*P* = 0.480(
Day 21	Group 1 (Glucantime^®^)	Most effective; significant difference vs. G2, G5, G7. ⁽²⁾	*P* = 0.0246 vs. G2; ***P < 0.0001 vs. G5 & G7
Day 21	Group 3 (AmB-loaded Nanofiber)	Significant reduction vs. control group.	*P* = 0.0081 vs. G7
Day 21	Group 3 vs. Group 2	No significant difference detected.	NS )*P* = 0.769(
Day 21	Group 5 & Group 7	Progressive lesion enlargement observed.	—

⁽¹⁾ Reflects the statistically superior response of Glucantime^®^ vs. blank nanofiber and untreated groups at Day 14

⁽²⁾ Glucantime^®^’s high efficacy persisted to Day 21 with stronger statistical significance vs. all comparator groups

##### Long-term lesion progression (Days 0–56)

**Fig. 10:**
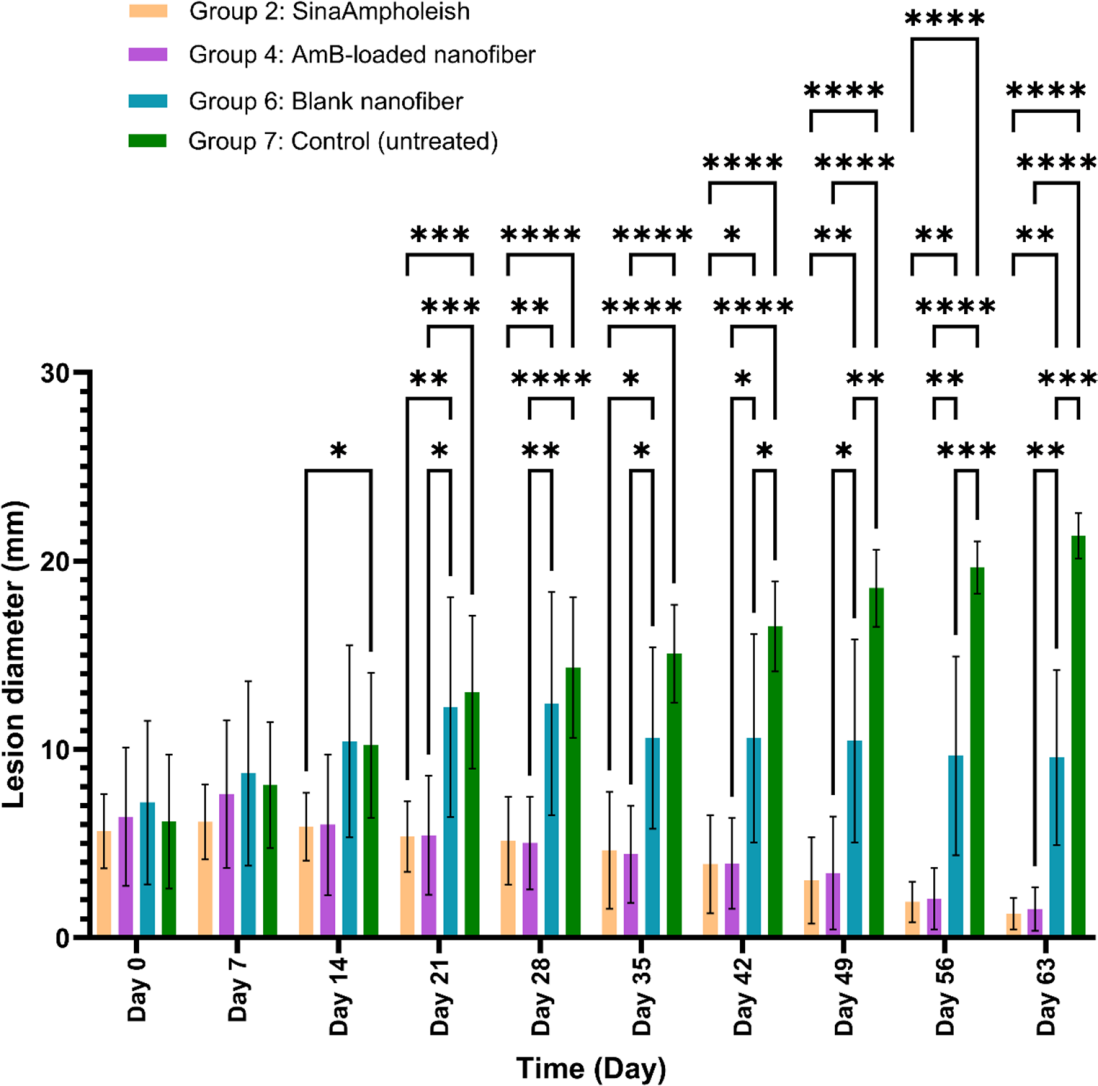
Lesion progression in long-term treatment groups (Groups 2, 4, 6, and 7) on Days 0, to 63. Weekly application of AmB-loaded nanofibers (Group 4) led to sustained lesion regression comparable to that of twice-daily SinaAmpholeish^®^ (Group 2). Minimal healing was observed in Group 6 (blank nanofibers), while lesions in the untreated control group (Group 7) continued to enlarge, highlighting the importance of active drug delivery

From Day 28, weekly administration of AmB-loaded nanofibers (Group 4) led to sustained and significant lesion size reduction compared to both blank nanofibers (Group 6) and the untreated control (Group 7) (*P* < 0.01). Group 4 showed no significant difference from SinaAmpholeish^®^ (Group 2), indicating comparable long-term efficacy. From Day 42, Group 6 also exhibited significant improvement over control, possibly due to barrier or hydration effects (*P* = 0.0374 on Day 42; *P* = 0.0006 on Day 63). On Day 63, both Groups 2 and 4 achieved marked lesion reduction versus control (*P* < 0.0001), supporting the potential of nanofiber-based systems as effective topical alternatives. Detailed outcomes are presented in (Table 5), and lesion progression over time is illustrated in (Fig. 10).

**Table 5:** Lesion size reduction from day 28 to 63. AmB-loaded nanofibers (Group 4) showed sustained efficacy vs. blank and control groups. No significant difference was found between group 4 and SinaAmpholeish^®^ (Group 2), indicating comparable long-term effectiveness

Day	Group	Outcome	*P*-value
Day 28	Group 4 (AmB-loaded Nanofiber)	Sustained and significant reduction vs. Group 6 (Blank) and Group 7 (Control).	*P* < 0.01
Day 28	Group 2 vs. Group 4	No significant difference observed; similar long-term efficacy.	NS )*P* = 0.999(
Day 42	Group 6 (Blank Nanofiber)	Statistically significant improvement over control group begins to emerge.	*P* = 0.0374
Day 63	Group 6 (Blank Nanofiber)	More pronounced lesion size reduction vs. control, possibly due to wound hydration.	*P* = 0.0006
Day 63	Group 2 (SinaAmpholeish^®^)	Marked reduction in lesion size vs. control group.	*P* < 0.0001
Day 63	Group 4 (AmB-loaded Nanofiber)	Marked reduction in lesion size vs. control group.	*P* < 0.0001
Day 63	Group 2 vs. Group 4	No significant difference; supports comparable efficacy.	NS (*P* = 0.956)

##### Estimated parasite load

Based on semi-quantitative lesion size scoring as a proxy for parasite burden (Khamesipour et al. 2022; Schuster et al. 2014) a progressive decrease in parasite load was observed in the Glucantime^®^-treated group and both groups receiving (AmB) formulations (nanofiber and SinaAmpholeish^®^). As presented in (Table 6), by Day 21, the parasite burden in these groups dropped from moderate (++) to low (+), while the control and blank nanofiber groups progressed to very high parasite levels (++++) consistent with uncontrolled infection. Similarly, in the long-term experiment, (Table 7) demonstrates that weekly application of AmB-loaded nanofibers (Group 4) and twice-daily SinaAmpholeish^®^ (Group 2) resulted in a significant reduction with respect to both controls in estimated parasite burden by Day 63. In contrast, the control and blank nanofiber groups showed fixed or increased lesion sizes, corresponding to high or very high parasite loads.

**Table 6:** Semi-quantitative Estimation of parasite burden in short-term treatment groups (Days 0–21) based on lesion diameter. Lesion size was categorized into four levels of parasite load: (+) low, (++) moderate, (+++) high, and (++++) very high. Glucantime^®^ treatment significantly reduced lesion size and estimated burden, whereas blank nanofibers and the untreated control showed notable progression of infection

Group	Day 0 Lesion (mm^2^)	Score	Day 21 Lesion (mm^2^)	Score	Parasite Burden Trend
G1 – Glucantime^®^ IP	~ 4.6	(++)	~ 2.75	(+)	Decreased significantly
G2 – SinaAmpholeish^®^	~ 5.7	(++)	~ 5.4	(++)	Stable/slight decrease
G3 – AmB-loaded nanofiber	~ 6.7	(++)	~ 5.8	(++)	Stable/slight decrease
G5 – Blank NF	~ 6.3	(++)	~ 10.8	(+++)	Increased significantly
G7 – Control	~ 6.2	(++)	~ 13.0	(++++)	Strong increase

**Table 7:** Estimated parasite burden in long-term treatment groups (Days 0–63) using lesion diameter as an indirect indicator. Weekly treatment with AmB-loaded nanofibers and twice-daily topical SinaAmpholeish^®^ markedly reduced parasite burden. In contrast, blank nanofiber and control groups demonstrated persistent or increased lesion sizes, suggesting ineffective or absent therapeutic impact

Group	Day 0 Lesion (mm^2^)	Score	Day 63 Lesion (mm^2^)	Score	Parasite Burden Trend
G2 – SinaAmpholeish^®^	~ 5.7	(++)	~ 1.3	(+)	Strong decrease
G4 – AmB-loaded nanofiber	~ 6.4	(++)	~ 1.1	(+)	Strong decrease
G6 – Blank NF	~ 7.2	(++)	~ 9.6	(+++)	Increased
G7 – Control	~ 6.2	(++)	~ 21.3	(++++)	Very strong increase

## Discussion

In this study, we explored the therapeutic efficacy of electrospun nanofibers composed of chitosan, gelatin, and PVA loaded with Amphotericin B (AmB) for the localized treatment of cutaneous leishmaniasis (CL) in BALB/c mice. Our results demonstrate that localized delivery via nanofiber patches not only accelerates lesion healing but also significantly mitigates the systemic toxicity and dosing limitations associated with free AmB administration. This aligns with previous reports supporting nanofiber-based drug delivery systems as promising alternatives in parasitic skin diseases due to their high surface-area-to-volume ratio, tailored drug release profiles, and improved tissue integration (Sill and Von Recum 2008; Xue et al. 2019). The nanofibers developed in our study exhibited uniform morphology and diameters below 200 nm, which are known to enhance transdermal drug penetration (Sill and Von Recum 2008). Incorporation of chitosan and gelatin improved the biocompatibility and structural integrity of the nanofibers, consistent with Mohebali et al.‘s findings using chitosan-based polyurethane nanocomposites loaded with meglumine antimoniate (MA) for topical CL therapy (Mohebali et al. 2025). Our drug release analysis revealed a biphasic pattern, with an initial burst followed by sustained release over 72 h, consistent with the controlled kinetics reported by Mahmoudi et al. for royal jelly/propolis-loaded nanofibers containing Glucantime^®^ (Mahmoudi et al. 2023). This controlled release profile likely contributes to both rapid parasite suppression and continued drug presence at the lesion site, which is essential for complete therapeutic efficacy. Importantly, the IC₅₀ values derived from MTT assays on HDF and THP-1 cells showed that AmB-loaded nanofibers were significantly less cytotoxic than free AmB, echoing the results of Riezk et al., who demonstrated reduced AmB toxicity via chitosan-based nanoparticle formulations (Riezk et al. 2020).

These findings may hold promise for future translational research, although additional validation is required before any clinical relevance can be inferred. Safety concerns have long limited the widespread use of AmB in cutaneous applications.

This is particularly important from a translational perspective, as safety concerns have long limited the widespread use of intravenous free AmB for cutaneous leishmaniasis, which is not approved for this indication in most countries. In this context, liposomal AmB (AmBisome^®^) has been shown to reduce systemic toxicity while maintaining efficacy, making it a more appropriate comparator for evaluating the performance of AmB-loaded nanofibers than free IV AmB. In vivo analysis further confirmed that the nanofiber patches achieved superior lesion reduction in both short-term (14-day) and long-term (56-day) treatment groups. A weekly application regimen (Group 4) demonstrated therapeutic results comparable to the twice-daily application of SinaAmpholeish^®^ (Alizadeh et al. 2023), suggesting improved practicality of use rather than making claims about patient adherence.

To the best of our knowledge, this is the first report of such a dosing regimen for AmB-loaded nanofibers in the CL mouse model, indicating a potentially useful approach for extended-release topical delivery, while further validation is needed.

The nanofiber patches used in this study were applied directly over the CL lesions in the murine model to maximize local drug exposure and promote parasite suppression. This mode of application ensures close contact between the drug-loaded fibers and the infected tissue, supporting sustained local release while minimizing systemic exposure. Although tail-base inoculation was used for practical reasons in this experimental model, whether similar lesion-targeted patch-based approaches could be applicable in clinical settings remains uncertain and requires further investigation. Any potential clinical use is speculative at this stage.

To contextualize the therapeutic effect observed in vivo, previously reported reference IC₅₀ values for meglumine antimoniate (MA) against promastigotes, amastigotes, axenic, and intracellular forms of L. major (3.8 ± 0.34, 9.5 ± 0.7, 311.5 ± 61, and 52.3 ± 8.4 µg/mL, respectively) provide useful benchmarks for comparison (Derakhshani et al. 2023; Pujals et al. 2008).

MA was selected as the positive control in this study because it is the first-line therapy for cutaneous leishmaniasis in most countries and is widely recognized as a clinical standard. The choice of MA over parenteral AmB was motivated by limitations in accessibility and systemic toxicity associated with injectable AmB, whereas the present study aimed to develop a safe, effective, and potentially translatable topical treatment. However, conclusions regarding clinical translation cannot be drawn from this murine model and require further investigation.

Furthermore, the group treated with blank nanofibers (without AmB) exhibited a delayed but measurable reduction in lesion size after 42 days, a finding supported by Mohebali et al., who reported that chitosan alone may aid wound healing and immunomodulation even in the absence of antileishmanial agents (Mohebali et al. 2025). This reinforces the dual functionality of chitosan as both a structural and bioactive material. Parasite load reduction that was approved by using NeoBar slides, confirmed the findings of Saberi et al., who had proven this through qPCR. These findings indicated a reduction in parasite burden that mirrored lesion resolution (Saberi et al. 2025). Unlike conventional intralesional therapies such as Glucantime^®^ that require daily and painful administration, nanofiber-based delivery in our study achieved therapeutic outcomes with fewer applications, although direct clinical comparison cannot be inferred. Finally, all experimental procedures followed ethical standards with humane endpoints and consistent lesion scoring methods, improving both reproducibility and translational relevance. In conclusion, our findings suggest that AmB-loaded electrospun nanofibers may represent a promising localized therapy for CL. Compared to previous approaches—such as liposomal AmB (SinaAmpholeish^®^), Epidermal Growth Factor (EGF) co-therapy, or chitosan–antimonial composites (Alizadeh et al. 2023; Mahmoudi et al. 2023; Saberi et al. 2025) —our system offers the advantages of extended release, biocompatibility, and reduced application frequency in the murine model, though clinical extrapolation will require further studies. Future studies may focus on optimizing patch size, extending release durations beyond 7 days, and evaluating human skin models to accelerate clinical translation.

## Limitations and future directions

Although this study demonstrated promising therapeutic outcomes, it has certain limitations. First, all experiments were performed in a murine model of acute cutaneous leishmaniasis, which may not fully recapitulate the human clinical response, where most CL cases tend to be chronic with long-term relapses. Future studies should consider evaluating the nanofiber platform in chronic or relapse-prone models to better simulate field conditions and assess its potential efficacy in such scenarios. Second, while our current nanofiber formulation loaded up to ~ 10 mg AmB per 550 cm², it remains to be determined whether higher loading capacities are feasible and whether this could further enhance the potency of the system. Although weekly application of AmB-loaded nanofibers produced therapeutic outcomes comparable to twice-daily SinaAmpholeish^®^, more frequent dosing (e.g., two nanofibers per week) was not explored in this study. Future investigations could evaluate alternative dosing schedules to generate additional comparative efficacy data. Optimization of drug loading without compromising fiber integrity or release kinetics represents an important area for future research. Moreover, immunological assessments such as cytokine profiling (e.g., IFN-γ, IL-10) were not performed, which could have provided valuable insights into host immune modulation. Future investigations should incorporate these evaluations to better understand the mechanism of action, as suggested by de Oliveira et al. (De Santana et al. 2022). Finally, broader comparative studies against other standard therapies, such as Miltefosine or alternative antileishmanial regimens, may further validate the advantages and limitations of the nanofiber platform, particularly in settings with antimonial resistance. Future evaluation could be considered if additional preclinical studies convincingly support its safety, efficacy, and overall performance, as noted by Roatt et al. (2020).

## Conclusion

This study demonstrates that electrospun chitosan/gelatin/PVA AmB-loaded nanofibers may represent a promising localized therapy for cutaneous leishmaniasis in the murine model. The nanofibers exhibited uniform morphology, mechanical stability, and a biphasic drug release profile that maintained therapeutic levels over 72 h. In vitro, they achieved up to 89.6% reduction in intracellular *L. major* amastigotes, with an estimated IC₅₀ corresponding to 1 × 1 cm² to 2 × 2 cm² patches, while free AmB completely inhibited parasites at ≥ 2.5 µg/mL. In vivo, weekly application of the AmB-loaded nanofiber patches reduced lesion size by 73.3% over 56 days, comparable to SinaAmpholeish^®^ and superior to blank or untreated controls. Cytotoxicity studies showed IC₅₀ values of 107.9 µg/mL (24 h) and 52.83 µg/mL (48 h) in HDF cells, with MIC₅₀ around 160 µg/mL and no detectable MIC₉₀. In THP-1 cells, IC₅₀ and MIC₅₀ were approximately 18.18 µg/mL (24 h) and 30 µg/mL (48 h), with MIC₉₀ estimated at 160 µg/mL after 48 h. These findings highlight the potential of AmB-loaded nanofibers to enhance therapeutic efficacy while minimizing cytotoxicity and reducing dosing frequency in preclinical (murine and in vitro) settings; however, clinical extrapolation will require additional preclinical and safety studies.

## Supplementary Information

Below is the link to the electronic supplementary material.

Supplementary file 1 (DOCX 32.0 KB)

## Data Availability

The datasets generated and/or analyzed during the current study are available from the corresponding author on reasonable request.

## Abbreviations

AmB: Amphotericin B
BALB/c: Strain of laboratory mice
CL: Cutaneous leishmaniasis
DMEM: Dulbecco’s Modified Eagle Medium
DMSO: Dimethyl sulfoxide
FBS: Fetal bovine serum
FTIR: Fourier-Transform Infrared Spectroscopy
HDF: Human dermal fibroblast
IFN-γ: Interferon-gamma
IL-10: Interleukin-10
IP: Intraperitoneal injection
IACUC: Institutional Animal Care and Use Committee
MTT: 3-(4,5-dimethylthiazol-2-yl)-2,5-diphenyl tetrazolium bromide
PBS: Phosphate-buffered saline
PMA: Phorbol 12-myristate 13-acetate
PVA: Polyvinyl alcohol
RPMI-1640: Roswell Park Memorial Institute medium 1640
SEM: Scanning Electron Microscopy
THP-1: Human monocytic cell line
WHO: World Health Organization
XRD: X-ray diffraction
