# Supplementary material for: Nanofibrous patches for targeted therapy of cutaneous leishmaniasis caused by Leishmania major: a preclinical amphotericin B platform
Source: Parasitol Res. 2025 Dec 11;124(12):160. doi: 10.1007/s00436-025-08605-x (PMC12698821; doi:10.1007/s00436-025-08605-x)
Supplement: Supplementary file 1 — (DOCX 32.0 KB) [file 436_2025_8605_MOESM1_ESM.docx]

**Table S1.** Breakdown of euthanized animals across groups according to predefined ethical endpoints

| Group | Treatment | Number of animals euthanized | Main reason(s) |
| --- | --- | --- | --- |
| 1 | Glucantime® (Positive Control) | 4 | Severe ulceration, weight loss, lethargy |
| 3 | AmB-loaded nanofiber (every other day) | 3 | Severe ulceration, lesion size >20 mm² |
| 5 | Blank nanofiber (every other day) | 3 | Severe ulceration, weight loss |
| 7 | Negative Control | 6 | Severe ulceration, lesion size >20 mm² |

**Table S2.** FTIR band positions (cm⁻¹) and assignments for individual components and AmB-loaded composite nanofibers

| Material | Key Peaks (cm⁻¹) | Functional Groups / Assignment |
| --- | --- | --- |
| Gelatin | 3300–3400, 1640, 1540, 1240 | O–H/N–H stretching, Amide I, II, III |
| PVA | 3280–3300, 2910, 1140, 840 | O–H, C–H, C–O–C, C–C |
| Chitosan | 3350, 1650, 1560, 1020–1150 | O–H/N–H, Amide I/II, C–O stretching |
| Amphotericin B | 3400, 1700, 1450, 1550, 1600, 1100–1200 | O–H, C=O, C=C polyene, C–O |
| Composite Fiber (AmB-loaded) | 3300, 1650, 1540, 1140 | Overlapping bands indicating hydrogen bonding and AmB entrapment |
